# Prospects and Challenges of Bacteriophage Substitution for Antibiotics in Livestock and Poultry Production

**DOI:** 10.3390/biology13010028

**Published:** 2024-01-04

**Authors:** Aoyu Jiang, Zixin Liu, Xiaokang Lv, Chuanshe Zhou, Tao Ran, Zhiliang Tan

**Affiliations:** 1 CAS Key Laboratory for Agri-Ecological Processes in Subtropical Region, National Engineering Laboratory for Pollution Control and Waste Utilization in Livestock and Poultry Production, Hunan Provincial Key Laboratory of Animal Nutrition Physiology and Metabolic Process, Institute of Subtropical Agriculture, Chinese Academy of Sciences, Changsha 410125, China; jiangaoyu21@mails.ucas.ac.cn (A.J.); liuzixin20@mails.ucas.ac.cn (Z.L.); zltan@isa.ac.cn (Z.T.); 2 University of Chinese Academy of Sciences, Beijing 101408, China; 3 College of Animal Science, Anhui Science and Technology University, Bengbu 233100, China; 13121191399@163.com; 4 College of Pastoral Agriculture Science and Technology, Lanzhou University, Lanzhou 730000, China; 5 State Key Laboratory of Herbage Improvement and Grassland Agro-Ecosystems, Ministry of Agriculture and Rural Affairs, Lanzhou University, Lanzhou 730000, China

**Keywords:** bacteriophage, antibiotics, antibiotic resistance genes, livestock

## Abstract

**Simple Summary:**

Bacteriophages, a class of viruses that exclusively infect bacteria, share a prolonged evolutionary history with their hosts. There are three life cycle modes including lytic, lysogenic, and chronic infection for bacteriophages. Lytic bacteriophages rely on the lysis cycle to reproduce continuously, lysogenic bacteriophages integrate their genes with the bacterial genome and transmit them with bacterial division, whereas chronic bacteriophages can replicate genetic material and assemble progeny bacteriophages without destroying the host cell structure. Currently, bacteriophages possess a plethora of applications and potential in human bacterial diseases and enteropathogenic diseases of livestock and poultry, specifically in the direction of antibiotic substitution, which exhibits tremendous potential for practical applications. Therefore, it is necessary to consider the benefits of bacteriophages in improving intestinal microecology and enhancing immune function, while also acknowledging the potential risks of instability in their application. To maximize their effectiveness at inhibiting antibiotic-resistant bacteria, the exploration of bacteriophages should be combined with cell culture, multi-omics, and genetic engineering technologies, and bacteriophage cocktails should be designed through careful consideration.

**Abstract:**

The overuse and misuse of antibiotics in the livestock and poultry industry has led to the development of multi-drug resistance in animal pathogens, and antibiotic resistance genes (ARGs) in bacteria transfer from animals to humans through the consumption of animal products, posing a serious threat to human health. Therefore, the use of antibiotics in livestock production has been strictly controlled. As a result, bacteriophages have attracted increasing research interest as antibiotic alternatives, since they are natural invaders of bacteria. Numerous studies have shown that dietary bacteriophage supplementation could regulate intestinal microbial composition, enhance mucosal immunity and the physical barrier function of the intestinal tract, and play an important role in maintaining intestinal microecological stability and normal body development of animals. The effect of bacteriophages used in animals is influenced by factors such as species, dose, and duration. However, as a category of mobile genetic elements, the high frequency of gene exchange of bacteriophages also poses risks of transmitting ARGs among bacteria. Hence, we summarized the mechanism and efficacy of bacteriophage therapy, and highlighted the feasibility and challenges of bacteriophage utilization in farm animal production, aiming to provide a reference for the safe and effective application of bacteriophages as an antibiotic alternative in livestock and poultry.

## 1. Introduction

With the continuous expansion of modern farming scale, the prevention and treatment of bacterial diseases in the process of intensive livestock and poultry farming has become an urgent problem to be solved. Traditionally, antimicrobial drugs, especially antibiotics, are used to inhibit the amplification of intestinal pathogens, promote the growth of animals, and improve the feed conversion rate [1]. However, the long-term use of antibiotics at high doses in animal husbandry has led to the disruption of gastrointestinal microbiota balance in livestock and poultry and aroused the emergence of resistance of pathogens to antibiotics. Whenever a novel type of antibiotic is developed and applied, bacteria with a specific resistance mechanism (e.g., enzyme inactivation, decreased intracellular concentration, or modification of target sites) will rapidly emerge [2]. Strikingly enough, available studies have shown the presence of at least 4043 antibiotic resistance genes (ARGs) in the rumen of ruminants, including beta-lactams (726 genes), glycopeptides (510 genes), tetracycline (307 genes), and aminoglycosides (193 genes) [3]. Similarly, it is reported that 97.3% of the 37 strains of *Escherichia coli* isolated from the rectum of diarrheic piglets are resistant to at least four distinct antibiotics, and 28 strains possess mucin resistance genes [4]. Notably, the growth-promoting and therapeutic effects of established antibiotics are gradually decreasing, and livestock products containing antibiotic residues are entering the human body, posing a serious threat to human health [5]. In this regard, China officially banned the addition of growth-promoting additives (including antibiotics) other than Chinese herbs to livestock and poultry feed in July 2020.

It is urgently required to replace feeding antibiotics with low-cost and high-efficiency alternatives to ensure the sustainable development of the intensive livestock and poultry industry, and numerous feed additives like plant secondary metabolites, probiotics, and acidifiers, have been developed and utilized to varying degrees. Natural phenolic compounds (NPCs), such as flavonoids, phenolic acids, stilbenes, and lignans, exist abundantly in plants. NPCs perform many biological functions, including regulating intestinal microbiota and maintaining the intestinal epithelial barrier; moreover, they have received widespread attention due to their purely natural, non-polluting, and low-residue characteristics [6,7,8]. Nevertheless, the isolation and purification of NPCs in nature are complex and expensive, and they tend to break down into oligomers in an aerobic environment. NPC-rich plants may hinder the digestion and absorption of nutrients in the gastrointestinal tract (GIT) due to the presence of antinutritional factors such as trypsin inhibitors and lectins [9]. As a representative strain of probiotics, the direct addition of *Bacillus sphaericus* to feed increases the average daily weight gain of weaned piglets and broilers, and reduces the incidence of post-weaning diarrhea of piglets by approximately 30%, which is comparable to that of antibiotic-treated groups, helping to minimize the abuse of antibiotics, and promoting the healthy growth of the livestock and poultry [10]. Nonetheless, the addition of probiotics may accelerate nutrient depletion in the gut, and the balance point of the intestinal microbiota is difficult to acquire. Some scholars have attempted gene editing of *Lactobacilli* using CRISPR-Cas technology, and even with the same genus of *Lactobacillus plantarum*, editing of the same locus in different strains has not consistently succeeded [11]. Acidifiers are used to improve nutrient digestibility by decreasing gastrointestinal pH, regulating bacterial microbiota structure, increasing the activity of protein hydrolysis enzymes, and extending nutrient digestion time. It was confirmed that acid mixtures are significantly more effective than organic acids or acid salts alone in improving pig performance [12]. Yet the effect of acidifiers is excessively one-sided, as it is easily neutralized by the alkaline substances in the feed, with limited inhibition of pathogens. As research continues, many alternatives for antibiotics have been exposed as drawbacks, with their effects usually limited by quality control, dosage, cost, palatability, and gastrointestinal environment, with prolonged use stimulating the evolution of pathogens and the development of ARGs [13].

As natural invaders of bacteria, bacteriophages have gained increasing attention for being used as effective alternatives to antibiotics. There is a long-standing competitive and symbiotic relationship between bacteriophages and bacteria. Unlike other antibiotic alternatives, bacteriophages are a special group of active organisms that automatically explore the host’s surface-specific receptors and reproduce the progeny bacteriophages while lysing the bacteria. Since bacteriophages evolve according to the weaknesses of their hosts, bacteriophages offer novel possibilities for solving the bacterial resistance challenge from the perspective of the species’ genes [14,15]. Based on the mutual evolutionary relationship between bacteria and bacteriophages, the phage resistance of bacteria has also been discovered [16,17]. Therefore, researchers have moved their attention to bacteriophages with the expectation of finding a breakthrough in replacing antibiotics using bacteriophages. This review summarizes the impact of bacteriophage substitution for antibiotics in livestock and poultry feeds, discusses its feasibility, and proposes targeted preventive measures to overcome bacterial bacteriophage resistance and instability, enhancing the safety of bacteriophage application.

## 2. Brief Introduction of Bacteriophages

Established studies have shown that bacteriophages, as a group of viruses that target specific bacteria, are not only widely distributed in the environmental niches (e.g., hydrothermal vents and sediments), but also present in surface lifeforms (e.g., animals and plants) [18,19]. The total estimated number of virus-like particles on Earth is approximately 10^31^, and their biomass is more abundant compared with prokaryotes [20,21]. Bacteriophages and bacteria essentially maintain a subtle symbiotic relationship. The genetic information of bacteriophages consists of four structural forms, including ssDNA, dsDNA, ssRNA, and dsRNA, which are highly adaptable to their environment. After entering into the host bacterial strain, bacteriophages determine the genetic information replication strategies according to different life cycle modes (lytic, lysogenic, and chronic) [15]. The virulent bacteriophages (like *T4* phage) can directly carry out DNA replication and protein shell synthesis, lysing the bacteria to release assembled bacteriophage progeny; the genome of the mild bacteriophages replicates synchronously with the host DNA either in a free plasmid state (like *P1* phage) or chimerically within the bacterial chromosome (like *λ* phage) and exits from lysogeny to a lytic state and starts assembling bacteriophage progeny in response to external factor induction. Whereas filamentous bacteriophages undergo chronic cycling (*Inoviridae* family, e.g., *Pseudomonas aeruginosa* phage *Pf* or *E. coli* phages *fd* and *M13*), during which progeny bacteriophages are released into the environment through the bacterial outer membrane instead of lysing the host [15]. Although the host avoids being lysed during chronic infection, their growth rate is greatly restricted. Direct lysis of bacteria by virulent bacteriophages is usually considered to be the only effective way to remove target bacteria, but it has been shown that genetic engineering techniques can actively remove partial or all of the deterrent genes of mild bacteriophages to make them exit from the lysogenic state and switch to the lytic state, which appears to be more efficient in the case of certain pathogens exclusively infected by mild bacteriophages (e.g., *Mycobacterium* spp.) [22].

Recently, researchers analyzed bacteriophages in livestock and poultry using metagenomics or viromics. There are 397,180 viral operational taxonomic units (vOTUs) present in the rumen of ruminants, and about 10.29% of vOTUs are predicted to be bacteriophages, 3.8% of which are able to infect multiple bacterial phyla, which is significantly higher than the proportion of bacteriophages that could infect multiple bacterial phyla in the human gut (0.13%) [23]. At the species level, the dominant bacteriophages in the feces of healthy piglets are *E. coli* phage *FV3*, crAssphage *cr116_1*, and *Enterococcus faecalis* phage *FP_oengus*, which greatly differ from the composition of the predominant bacteriophages in diarrheic piglets [24]. The feces and liver of chickens include a similar viral composition, with 10^3^–10^6^ bacteriophages/g present in the liver, and these bacteriophages predominantly infect *E. coli* [25]. These studies constitute an initial exploration of bacteriophages in the GIT and organs of livestock and poultry, but our knowledge of the species composition and life activities of bacteriophages, both in animals and farm environments, is still extremely limited.

## 3. Application of Bacteriophage in Substituting Antibiotics in Livestock and Poultry Production

Within the past two decades, bacteriophage research and development centers have been established in China, the United States, and the European Union, and the specifications and guidelines for bacteriophage application in industry have been continuously developed. In order to fill the gap in regulations and standards for bacteriophage clinical application, the China Phage Research Alliance has published a standard document on bacteriophage therapy for the first time, which further refines the operational procedures and technical requirements [26]. In livestock and poultry production applications, the European Medicines Agency formally approved the world’s first scientific guideline on bacteriophage veterinary medicines two months ago, *Quality, safety, and efficacy of bacteriophages as veterinary medicines—Scientific guideline* [27]. The guideline defines bacteriophage processing as a novel therapy, focuses on the safety and residue testing of bacteriophage therapies, and imposes specific requirements for animal clinical trials. It is evident that bacteriophage therapy has demonstrated a highly strategic importance in the livestock and poultry industry.

Compared with other antibiotic alternatives, bacteriophages possess several irreplaceable advantages. Firstly, bacteriophages are more diverse, with greater selectivity and operability; secondly, the target point of phage action is more precise, which can rapidly adsorb and kill the host bacteria, and will not disrupt the normal bacterial balance in the body; thirdly, since bacteriophages communicate with host genes to promote co-evolution, strategically designed bacteriophage cocktails could effectively prevent bacterial resistance; and finally, bacteriophages cannot multiply in eukaryotic cells and therefore generate no direct pharmacological effects in animals (unlike cytokines, hormones, and autoantibodies), leaving no mechanism-based concerns about toxicity in animals [28,29]. In practice, bacteriophages are applied in the form of bacteriophage cocktails, which refers to the mixture of multiple bacteriophages or the supplementation of multiple types of bacteriophages at different time points, in order to prevent bacteria from developing resistance to a single bacteriophage variety. Bacteriophage cocktails involve the combination of multiple bacteriophage invasions and lysis mechanisms targeting the same bacterial hosts and their relatives to achieve the best efficacy [22,30].

Bacteriophages have been demonstrated to be effective in replacing feed antibiotics in livestock and poultry (Table 1). The addition of 400 mg/kg of a bacteriophage cocktail (targeting *Salmonella*, *E. coli*, *Clostridium perfringens,* and *Staphylococcus aureus*) to weaned piglet diets significantly increased the average daily feed intake, improved the integrity of the intestinal barrier, and prevented the infiltration of pathogens into the intestinal cells, thus inhibiting intestinal inflammation and reducing the rate of diarrhea in piglets [31]. Supplementation of 1.5 g/kg of a bacteriophage cocktail to weaned piglet diets significantly increased the abundance of ileal *Lactobacillus* spp. and reduced the content of *E. coli* and *Clostridium difficile* in the ileum by 5.76% and 4.21%, respectively [32]. *L.* spp. reduces the pH in the intestinal tract via anaerobic fermentation, which further suppresses the growth of pathogens and maintains the stability of intestinal microecology [32]. Furthermore, the supplementation of feed with bacteriophages linearly increases mean daily weight gain and apparent digestibility in growing pigs and significantly increases the relative abundances of *Bifidobacterium* and *Lactobacillus* in feces, as well as significantly reduces levels of *C. perfringens* and *E. coli* in feces [33].

In the “kill the winner” theory, bacteriophages prey on highly abundant bacterial hosts to regulate the microbiota and maintain the local microecological diversity to enhance resistance to pathogen invasion [34]. Thus, bacteriophages at specific titers help suppress pathogen amplification and maintain gastrointestinal microbial homeostasis in livestock and poultry suffering from gastrointestinal-related diseases. In a *Salmonella* challenging chicken model, substituting antibiotics with bacteriophage cocktails (*vB_SenM-2*, *vB_Sen-TO17*) not only slowed down the fluctuation of hematological parameters such as erythrocyte counts and erythrocyte pressure water balance in the organism caused by antibiotics but also restored plasma glutamic acid aminotransferase activities to normal physiological levels, which suppresses the growth of the pathogen and alleviates the metabolic pressure on the liver simultaneously [35]. *Salmonella* concentrations in the cecum and feces of laying hens decreased significantly with increasing concentrations of supplemental bacteriophages in the feed, and 10 mg/kg of bacteriophages led to 25.58% and 14.74% reductions in *Salmonella* counts in the cecum and feces, respectively [36]. Fecal microbes of broilers who received bacteriophage cocktails (500 mg/kg in feed, DM bases) were not different from the control group; however, significantly higher relative abundances of ileal *Bifidobacterium*, *Prevotella*, and *Lactobacillus salivarius* were observed. A lower relative abundance of *Lactobacillus aviarius* was observed compared with the antibiotic group [37].

Research on the addition of bacteriophages to ruminant feeds is relatively rare, probably owing to the huge number of microbes inhabiting the rumen. Microbes such as bacteria, fungi, and archaea assist ruminants in degrading carbohydrates for absorption and simultaneously provide a “natural medium” for bacteriophages to reproduce and evolve. Supplementation with bacteriophages through feeding exerts a minimal effect on rumen microbial structure, but modulating the structure of cattle diets alters the diversity of the rumen microbiota (antimicrobial resistance reservoirs), with the diversity and abundance of ARGs from the rumen of beef cattle on the high concentrate diet (concentrate:forage = 9.2:0.8) being significantly increased compared with those in the control group (concentrate:forage = 5:5) [38].

**Table 1:** Effects of dietary supplementation with different types and concentrations of bacteriophages on livestock and poultry.

Bacteriophage	Model	Effects	References
Mixture 1*	Weaned piglet	An amount of 400 mg/kg of bacteriophage mixture supplement significantly elevated villi height (VH)/crypt depth (CD) ratio (duodenum, jejunum, and ileum), significantly increased the relative mRNA expression of ZO-1, Claudin-1, and Occludin, and significantly decreased interleukin-1β and tumor necrosis factor-α in serum, as well as reduced diarrhea incidence.	Zeng et al. [31]
Cocktail 2*	Weanling pig	An amount of 1.0 g/kg of bacteriophage cocktail supplement significantly increased average daily gain, ileal *L.* spp. and villus height (duodenum and jejunum), and decreased relative abundance of *Coliforms* and *Clostridium* spp. (ileum).	Kim et al. [32]
Cocktail 3*	Barrows	An amount of 1.0 g/kg of bacteriophage cocktail supplement increased the average daily gain by 10.58%. With the increase in bacteriophage dose (0, 0.5, 1.0, and 1.5 g/kg), the abundance of *Bifidobacterium* spp. and *L.* spp. increased linearly, and the abundance of *C.* spp. and *Coliforms* decreased linearly in feces.	Kim et al. [33]
Cocktail 4*	Broiler chicken	An amount of 400 mg/kg of bacteriophage cocktail supplement significantly increased the relative abundance of *Bifidobacterium*, *Prevotella,* and *L. salivarius*; the relative abundance of *L. aviarius* decreased compared with antibiotic treatment (ileum).	Upadhaya et al. [37]
Cocktail 5*	Broiler chicken	An amount of 1.5 g/kg of bacteriophage cocktail supplement significantly increased the abundance of *Lactobacillus* (ileum), the serum concentrations of propionate (cecum), VH/CD, the serum concentrations of total antibody, immunoglobulin M (Ig M), and IgG, and thus significantly decreased the abundance of *Coliform* bacteria compared with the control group.	Sarrami et al. [39]
Mixture 6*	Laying hen	An amount of 10 mg/kg of bacteriophage mixture supplement significantly decreased the colonization of *Salmonella typhimurium* (spleen, oviduct, caecum, and excreta) and the mRNA expressions of IFNγ, HSP-27, and TNF-α compared with antibiotic treatment (liver).	Lee et al. [36]

1* A mixture of individual bacteriophages specifically targeting *Salmonella* (*Salmonella choleraesuis*, *S. derby*, *Salmonella dublin*, *S. enteritidis*, *Salmonella gallinarum*, *Salmonella pullorum*, and *S. typhimurium*), *E. coli* (K88, K99, 987P, F18, F41, and O78), *C. perfringens* (Type A, B, C, D, and E), and *S. aureus*. The concentration of individual bacteriophage in the mixture was 1.0 × 10^8^ plaque-forming units per gram (pfu/g).

2* Bacteriophages isolated from water, soil, and farm waste samples, which infect *Salmonella* (*S. typhimurium*, *Salmonella enteritidis*, *Salmonella cholerasuis,* and *Salmonella derby*), *S. aureus*, *E. coli* (k88, k99, and f41), and *C. perfringens* types A and C at the concentrations of 1.0 × 10^9^ pfu/g.

3* A mixture of bacteriophages targeting *Salmonella* (*S. typhimurium*, *S. enteritidis*, *S. cholerasuis,* and *S. derby*), *S. aureus*, *E. coli* (k88, k99, and f41), and *C. perfringens* types A and C at the concentrations of 1.0 × 10^9^ pfu/g.

4* A mixture of bacteriophages targeting *S. gallinarum*, *S. typhimurium*, *S. Enteritidis*, and *E. coli* at the concentrations of 1.0 × 10^8^ pfu/g each and *C. perfringens* (1.0 × 10^6^ pfu/g).

5* A mixture of several bacteriophages (2.04 × 10^8^ pfu/g) against *Salmonella* and *E. coli*.

6* Isolated from various samples (the sewage and excreta of several chicken farms in Korea) with bacteriolytic activity against *S. typhimurium* at the concentrations of 1.0 × 10^8^ pfu/g.

## 4. The Feasibility of Bacteriophages in Substituting Antibiotics

### 4.1. Regulation of Intestinal Microecology

**Figure 1:**
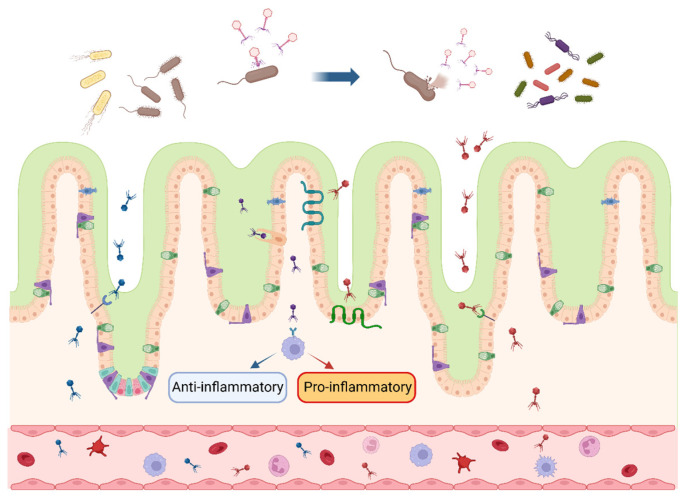
Interactions between bacteriophages and intestinal bacteria and cells. After multiplying and expanding within the intestinal host bacteria, bacteriophages secrete lytic enzymes to lyse the cell wall and release bacteriophage progeny, which continue to inhabit the host bacterial population. Bacteriophages enter the intestinal wall in response to specific receptors and cytophagy in intestinal epithelial cells and cross into the blood circulation, inducing anti-inflammatory (blue arrow) or pro-inflammatory (red arrow) response. The blue arrow at the top of the picture indicates that the bacteriophages infest and lyse the bacteria in the intestinal lumen. Different colors represent various species of bacteriophages and their progeny. (figure created using BioRender, https://biorender.com. accessed on 27 November 2023).

Gastrointestinal microbes play a pivotal role in the digestion and absorption of nutrients and in the construction of intestinal immune barriers; the distribution and function of bacteriophages in the intestine are shown in Figure 1. Countless bacteriophages and their host bacteria exist in the GIT of farm animals, and in the long-term struggle and coexistence state, bacteria and bacteriophages have evolved a variety of mutualistic mechanisms. On the one hand, besides host bacteria, virulent bacteriophages can exert indirect effects on non-host bacteria, thus exerting contact-dependent antagonistic effects on diverse Gram-positive bacteria [40]. Virulent bacteriophages are able to cause changes in the structure of intestinal microbiota through a cascade effect, which in turn affects the composition of intestinal metabolites (tryptamines, carbohydrates, nicotinamide mononucleotides, etc.), reduces neurotransmitter production, and alters bile acid metabolism, which finally leads to physiological and metabolic effects on the organism [41]. On the other hand, bacteriophages possess strong permeability, dynamic adaptability, and excellent biocompatibility, making them capable of crossing the multi-layered mucosal barrier and regulating the microbial community through the metabolic cycle in the body, thereby overcoming the deficiencies of antibiotics [42]. Bacteriophage supplementation in broiler feed restores fecal microbiota composition to normal levels, in contrast to the antibiotic group (reduced relative abundance of Firmicutes at the phylum level and *Lactobacillus* at the genus level) [37]. Similar results are observed in weaned piglets, where bacteriophages significantly affect bacterial diversity and intestinal metabolism by regulating the dominant composition of microbes. Compared with the antibiotic group, the addition of bacteriophages in feed significantly increases the microbial abundance in the cecum of piglets, exerting an environmental selective pressure on intestinal pathogens, which is crucial for maintaining gastrointestinal homeostasis [31].

### 4.2. Targeted Inhibition of Pathogens

Unlike the broad-spectrum inhibitory approach of antibiotics, bacteriophages exert host specificity for they can recognize specific signal molecules such as polysaccharides and proteins on the bacterial surface [43]. Bacteriophages are capable of encoding depolymerase to degrade macromolecules such as capsular polysaccharides, extracellular polysaccharides, and lipopolysaccharides (LPSs) on the outer surface of bacteria, thereby breaking through the bacterial external barrier, which accelerates the injection of bacteriophage nucleic acids into the bacteria to complete the invasion process [44].

The reasonabledesign of bacteriophage cocktails can accelerate the advancement of bacteriophages in replacing antibiotics for treating pathogen-induced diseases. Bacteriophage therapy is currently used to treat GIT diseases such as diarrhea induced by *E. coli* and *Salmonella* in pigs and poultry. *Enterococcus,* which has strong antibiotic resistance, is prevalent in the GIT of animals where it maintains a high abundance and aggravates the inflammatory response when treated with antibiotics. Both single bacteriophage and bacteriophage cocktails could be used to inhibit *Enterococcus* growth. However, a consistent proportion of mutations in the Epa extracellular polysaccharide synthesis gene of *Enterococcus* is observed when using a single bacteriophage, contributing to the prevention of initial recognition and adsorption of *Enterococci* by bacteriophages. Whereas co-treatment of *E. faecalis* with *Myoviridae*, *Siphoviridae,* and *Podoviridae* bacteriophages reduces the abundance of *E. faecalis* to about 10% of the normal growth state and effectively inhibits the growth of bacteriophage-resistant mutants [45]. Similarly, *S. typhimurium* can adhere to and invade intestinal epithelial cells. In both normal and *S. typhimurium*-infected intestinal epithelial cells INT-407, bacteriophage *P22* significantly reduces the amount of adsorption and infiltration of INT-407 by *S. typhimurium* [46]. Additionally, bacteriophage P22 induces the expression of pro-inflammatory genes in macrophage-like HD11 cells through a non-TLR-mediated recognition pathway, enhancing the immune response to kill *S. typhimurium*. Saez et al. [47] found that direct feeding of microencapsulated bacteriophages significantly reduced the detection of *S. typhimurium* 2 h and 4 h post challenge, compared with the gavage and control groups. Higher concentrations of anti-*Salmonella* bacteriophages were detected in the contents of the ileum and cecum in the treatment group than in the control group, suggesting that the bacteriophages successfully colonized the pig intestinal tract and exerted their bactericidal effects. Not coincidentally, Seo et al. [48] observed that *Salmonella* bacteriophage mixtures reduced the level of *Salmonella* detection in the feces of weaned piglets, demonstrating that bacteriophages offer an alternative to antibiotics for the treatment of *Salmonella* infections in swine. *E. coli*, a common Gram-negative pathogen, is susceptible to induce acute mastitis in early lactation of dairy cows and diarrhea in weaned piglets. In a cow model of strongly drug-resistant *E. coli*-induced mastitis, injection of a combination of bacteriophages (*vB_EcoM_SYGD1*, *vB_EcoP_SYGE1*, and *vB_EcoM_SYGMH1*) into the mammary glands of dairy cows significantly decreased the bacterial and somatic cell counts of the milk and significantly reduced the concentrations of IL-1β and TNF-α concentrations in the blood [49]. Similar results have been reported in other animal experiments. When weaned piglets were supplemented with 1 g/kg of a bacteriophage cocktail for 35 days, the abundances of *E. coli* and *C. difficile* in the ileum were significantly decreased compared with the control group, and the growth of pathogens was restricted; meanwhile, the bacteriophage cocktail was favorable to the predominance of probiotics such as *Lactobacillus*, with the digestive and absorptive functions of the intestine on nutrients enhanced, which notably increased the daily weight gain of the weaned piglets [32]. In a previous study, a bacteriophage cocktail containing phages *B44/1* and *B44/2* was reported to treat nearly 93% of calves with enterotoxigenic *E. coli* strain O9:K30.99-induced enteritis, significantly reducing the morbidity and mortality of the calves [50]. The above studies suggest that supplemental bacteriophage cocktails are capable of reversing the pathology induced by the pathogenic bacteria to achieve the equivalent effect of antibiotic treatment without the concern of antibiotic residuals, etc. These pilot studies have greatly strengthened our confidence in the application of bacteriophages as an alternative to antibiotics.

It is noteworthy that the LPS mutant evolved in *E. coli* to evade the bacteriophages show decreased resistance to tetracycline and mucomycin compared with the wild-type parental strain, and this effect persists throughout the 10-day test cycle in which the bacteriophages co-existed with *E. coli*. Mutated pathogenic bacteria lose their virulence while evading bacteriophages, which provides a novel insight for the application of bacteriophages in the treatment of livestock and poultry diseases [51]. The bacteriophages are able to maintain the health of intestinal microecology and promote the normal development of animal intestinal tissues, which is essential for the healthy growth and development of animals. Nevertheless, the complex gene regulatory network among phages, pathogenic bacteria, and organisms in disease states needs to be explored in more intensive in vivo research.

### 4.3. Mediation of Immune Response

The effectiveness of bacteriophages on livestock and poultry also relies on the interaction of bacteriophages with the body’s immune cells. Under intestinal homeostasis, bacteriophages in the intestinal lumen can pass through the intestinal epithelium into the lamina propria through specific receptor uptake by epithelial cells or specific cytophagy, and then migrate via the blood into the circulation to interact with the immune cells to induce a pro-inflammatory or anti-inflammatory response. This is accomplished with inhibited synthesis of NF-κB, IL-2, TNF-α, and IL-10 in the bloodstream, which play an indispensable role in the maintenance of immune responses of the animal system [52]. Simultaneously, diverse bacteriophages possess rapid and directional transfection ability in the confluent cell layer of the intestine, brain, lung, liver, and kidney, and traverse the epithelial cell layer within 10 min. For instance, the T4 phage maintains the apical-to-basal direction to cross the confluent cell layer for transport under the cytotropic effect of the Golgi apparatus. It is speculated that bacteriophages could enter the metabolic cycle of the organism from the GIT and then be transferred and exchanged among the organs of multiple systems [53].

The mucosal immune system is the primary barrier against pathogen invasion. The intestinal mucosal immune system is activated when the intestinal barrier structure is impaired, thereby initiating an immune response. It is well known that bacteriophages are usually enriched at the site of bacterial colonization, and countless bacteria in the gut provide the conditions for bacteriophages to modulate the organism’s immune response. Barr et al. [54] demonstrated that the number of bacteriophages adhering to the cell-associated mucus layer and mucin glycoprotein is 4–5 times higher than that of the non-mucosal layer. The bacteriophages’ adherence to the mucus model suggests that the immunoglobulin-like structural domains on the bacteriophage coat protein could interact with the mucin and surface glycoproteins of the epithelial cells, allowing bacteriophages to adhere tightly to the mucus layer, providing immunity from non-host sources and strengthening the mucosal defense barrier [54]. Combining metagenomic, meta-transcriptomic, and meta-viromic analyses, Yan et al. [55] found that 24.6–40.4% of shared viral populations exist in the mucosal–luminal interface of the colonic lumen versus feces; although there is heterogeneity in the local inflammation in IBD patients (healthy proximal colon versus inflamed distal colon), the distal and proximal colonic mucosal viral differences are significantly less than colon versus feces, suggesting that the colonic mucosa provides a different microecological environment for intestinal viruses compared with feces. It was reconfirmed that the tight binding between the bacteriophage coat protein and mucin promotes bacteriophage colonization in the intestinal mucosal layer and the construction of a microecological environment with a high degree of similarity [55]. These findings suggest that the symbiotic relationship between bacteriophages and the organism provides the intestinal mucosa with an invisible protective barrier against pathogens.

The application of bacteriophages enhances animal immunity in livestock and poultry production as well. In weaned piglets, bacteriophages activate the immune system by targeting the TLR-mediated inflammatory response in the intestinal mucosa, significantly increasing the concentrations of sIgA and TGF-α in the ileal mucosa and the mRNA expression of TLR-2, TLR-4, and TLR-9 in the jejunal mucosa [31]. Challenging laying hens with *Salmonella* activates the immune response to produce large amounts of pro-inflammatory cytokines and stimulates compensatory lesions and proliferation in the liver and spleen; however, the addition of 0.1% of bacteriophages to the *Salmonella*-challenged feed reduces the relative expression of TLR-4 in the jejunum, which is the main receptor for LPSs produced by Gram-negative bacteria, suggesting that bacteriophages effectively inhibit the growth and reproduction of *Salmonella* and reverse the immune imbalance in laying hens [36]. Nevertheless, the induction of pro-inflammatory or anti-inflammatory immune responses in livestock and poultry by bacteriophages and the detailed mechanism of bacteriophages with different bacterial hosts need to be further explored in order to provide a feasible reference for the maintenance of organismal immune homeostasis under pathological conditions.

### 4.4. Improvement in Intestinal Morphology

The GIT, as the largest immune organ in mammals, is characterized by microbial communities that determine the development of the immune system. Therefore, direct or indirect interactions between bacteriophages and microbiota in the intestine play an essential role in the health status of the intestine, including the intestinal morphology and structure. Villi height (VH), crypt depth (CD), and the VH/CD ratio have been commonly used to reflect intestinal health.

Pilot studies have shown that supplementation of bacteriophages in livestock and poultry feeds could improve intestinal morphology and structure, thus promoting the healthy development of the intestine. In a trial of bacteriophage supplementation to replace antibiotics in the feed of weaned piglets, Zeng et al. [31] demonstrated that the supplementation of bacteriophages in the basal feed at 400 mg/kg significantly increased the VH of the jejunum and ileum, decreased the jejunum and ileum CD, and significantly elevated the relative expression of mRNAs related to tight junction (ZO-1, Claudin-1, and Occludin) in the jejunum. With the effects of probiotic bacterial components and metabolites, the intestinal tissues develop in a homeostatic state, and it is extremely difficult for pathogens to break through the immune barrier composed of intestinal epithelial cells and bacteriophages; therefore the intestinal tract maintains a normal tissue morphology. Lee et al. [56] verified that the bacteriophage cocktails added to the feed of weaned piglets increased intestinal secretion of metabolites such as short-chain fatty acids by beneficial bacteria that regulate intestinal morphogenesis and significantly increased VH of the duodenum and jejunum. Similarly, Hosseindoust et al. [57] reported that bacteriophage cocktails improve the intestinal organization and microbial communities of weaned piglets, effectively enhancing the efficiency of digestion and absorption to promote overall body health. Zhao et al. [58] proved that the supplementation of 1.0 × 10^9^ PFU bacteriophages and 1 mg/mL of amoxicillin in a chick gavage experiment 8–10 days post hatch, respectively, significantly increased the jejunal VH and VH/CD ratio, but bacteriophage supplementation significantly increased the relative mRNA expression of jejunal Occludin and ZO-1. This result suggests that bacteriophages can interrupt the pathways of pathogens through the intestinal epithelium into the bloodstream, which exerts an important effect in maintaining intestinal microbiology and immune homeostasis.

Collectively, the integrity of the intestinal barrier is crucial for maintaining the nutrient absorption and immune function of intestinal epithelial cells. Dietary addition of bacteriophages is available to enhance the physical and microbial barrier function of the GIT and block the pathway for pathogens entering the circulatory system in the intestinal lumen; however, the specific mechanisms of bacteriophages need to be further explored.

## 5. The Challenge of Bacteriophage Application

### 5.1. The Battle between Bacteriophage and Bacteria

The mechanism of interaction between bacteriophage and bacterial communities has been reported in extensive studies. After evolving over long periods, both bacteriophages and bacteria have evolved in vivo systems for mutual offense or defense, focusing on three processes: bacteriophage adsorption, bacteriophage DNA injection, and cleavage of the bacteriophage genome [15,59,60].

The tail structures of bacteriophages help them recognize the surface receptors of specific bacteria and attach to the bacterial surface to initiate the process of infestation [61]. Mechanisms by which bacteria block bacteriophage adsorption include mutations in bacterial surface receptors and secretion of substrates that compete with bacteriophages for receptor binding sites. For example, *E. faecalis* can mutate polysaccharide antigens to block bacteriophage adsorption, and *Pseudomonas* can secrete exopolysaccharides to compete with bacteriophages for bacterial surface receptors, protecting the bacteria from bacteriophage infestation [43,62]. In the second phase of bacteriophage infestation, bacteria are able to block the entry of bacteriophage DNA into the bacterial interior through the Superinfection Exclusion (Sie) system. The *E. coli* bacteriophage *T4* encodes immunity proteins and spackle periplasmic proteins, which form the Sie system that inhibits the injection of bacteriophage DNA into the bacterium and prevents subsequent invasion by other T-even-like phages [63]. Furthermore, bacteria recognize and cleave the bacteriophage genomes through restriction–modification systems and the CRISPR-Cas system to inhibit their amplification [64,65]. Bacteria acquire resistance to bacteriophages by resisting bacteriophage infestation in the above ways, and some scholars have expressed concerns about bacteriophage therapy. A bacteriophage-resistant variant of *Acinetobacter baumannii TP3* has been isolated from patients with *Acinetobacter baumannii* infection even after administration of bacteriophage therapy [16]. In addition, *Klebsiella pneumoniae* and *Pseudomonas aeruginosa* have also been reported to exhibit bacteriophage resistance within the fifth and seventh days of bacteriophage treatment, respectively [17,66].

In response, bacteriophages have evolved the ability to recognize new receptors. For instance, the receptor-binding protein J of the bacteriophage *λ* is able to change its terminal structure to bind to the new receptor OmpF under the inhibited expression of the host surface receptor LamB [67]. In order to evade DNA cleavage by bacteria, bacteriophages have also evolved various defense mechanisms. On the one hand, bacteriophages could spontaneously mutate restriction endonuclease (REase) to recognize the receptor or methylate their own genomes with host Methyltransferase to avoid REase recognition [68]. On the other hand, the anti-CRISPR proteins carried by bacteriophages suppress the Csy complex from binding to the bacteriophage target DNA, thus protecting the bacteriophage genes [69]. These findings refresh our understanding of the relationship between bacteriophages and bacteria. We are encouraged because Erin et al. [70] screened *Pseudomonas aeruginosa BWHPSA011* containing the natural functional cyclic-oligonucleotide-based antiphage signaling system (CBASS), which can produce 3′,3′-cGAMP to activate effector proteins downstream of CBASS, thereby resisting bacteriophage *PaMx41* infestation. An anti-CBASS protein, *Acb2*, has been detected in an escape mutant of the bacteriophage *PaMx41* that inactivates it by binding 3′,3′-cGAMP, effectively disrupting bacterial CBASS immunization [70]. Interestingly, bacteriophages have also been recently reported to encode a small non-coding RNA anti-CRISPR that specifically interacts with *Cas6f* and *Cas7f*, strongly inhibiting the type I-F CRISPR-Cas system and leading to the formation of abnormal Cas subcomplexes [71]. This suggests that the anti-CRISPR technology could serve as a safety switch to regulate the CRISPR-Cas system, which could help improve the safety and therapeutic efficacy of the CRISPR-Cas system when used for genome editing and bacteriophage therapy. Additionally, bacteriophages and their metabolites, which exhibit superb bactericidal capabilities, have provided us with new ideas. As bacteriophage-encoded and secreted enzymes, endolysins hydrolyze the host cell wall from the bacterial interior and subsequently release bacteriophage progeny [72]. Jun et al. [73] showed that *SAL200,* featuring bacteriophage endolysins *SAL-1* as the active pharmaceutical ingredient, led to no production of staphylococcal mutants, and it was further found that no production of resistant mutants was observed in *Staphylococcus aureus* when exposed to the lowest inhibitory concentration of *SAL200*. Bacteria are unable to respond to bacteriophages in an extremely short time, which greatly reduces the probability of bacteria developing bacteriophage resistance [74]. Therefore, in order to weaken pathogen resistance to bacteriophages, we are supposed to look for weaknesses of bacteriophage resistance from the molecular structure to maximize the beneficial effects of bacteriophage therapy.

### 5.2. Challenges of ARG Diffusion

One of the key challenges in treating bacterial diseases is overcoming bacterial antibiotic resistance since bacteria restrict the entry of antibiotics by down-regulating the expression of pore proteins to reduce the permeability of the outer membrane. More importantly, bacterial genes encoding antibiotic targets can undergo single-point mutations that prevent them from binding antibiotics, and some genes encode synthetic degradation enzymes that decompose intracellular antibiotics [75]. These pathways offer great possibilities for bacteria to develop antibiotic resistance and further spread ARGs from different bacterial populations via horizontal gene transfer. The ARGs are detected in lots of farm animals, with 10^3^–10^6^ bacteriophages/g present in chicken liver carrying a large number of ARGs (e.g., *blaTEM*, *blaCTx-M-1*, *sul1*, *qnrA*, *armA*, and *tetW*), which are most likely to be transferred from the intestine to the liver, constituting a potential reservoir for the ARGs [25]. Naturally, the abundance of bacteriophages carrying ARGs may be overestimated or misclassified due to possible bacterial contamination during sampling. It has been shown that ARGs are also found in the rumen, and these ARGs can be used as potential biomarkers for health risk assessment of the rumen microbiome [38,76].

**Figure 2:**
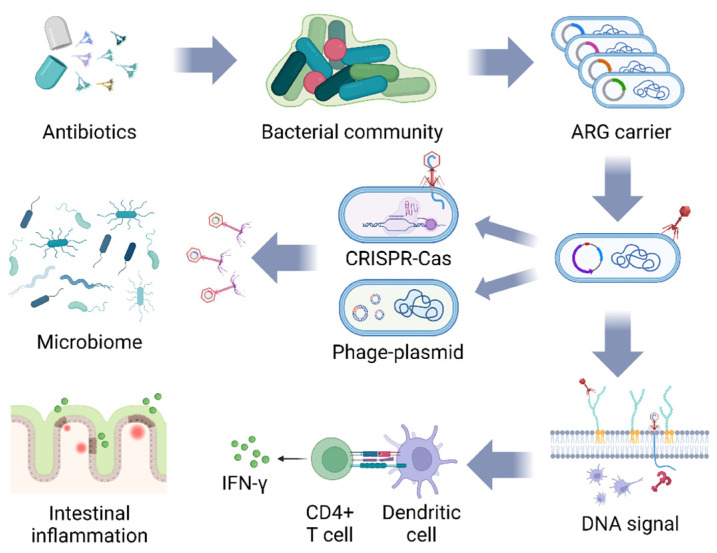
The potential threat of bacteriophage-mediated ARG diffusion and induction of IFN-γ synthesis. The bacterial population mutates to generate ARGs under the stimulation of antibiotics, and bacteriophages carrying ARGs bred from the previous host continue to infect the next group of bacteria, which is endowed with ARGs by means of bacterial CRISPR-Cas shearing and gene integration of bacteriophage plasmids, and repeated cycles lead to more microbial populations carrying ARGs. Bacteriophage DNA enters the bacteria and is recognized by TLR9, which stimulates dendritic cells to deliver antigen to CD4+ T cells, inducing them to synthesize IFN-γ to aggravate the intestinal inflammatory response (figure created in BioRender, https://biorender.com. accessed on 12 December 2023).

The diffusion pathways of ARGs between bacterial populations via bacteriophages are shown in Figure 2. A few studies report that bacteriophages promote the pathway of ARG transduction and diffusion among bacterial communities. When bacteriophages pierce their tails inside the bacteria and inject nucleic acids, the CRISPR-Cas system carried by the bacteria themselves specifically recognizes phage-derived sequences and inserts them into CRISPR-spacers [77]. Additionally, bacteriophages in the mild state can integrate their own gene sequences into the bacterial genome to form prophage, which diffuses along with the DNA replication of the host [15]. The auto-transduction process of lysogenic bacteriophages increases host bacterial survival by suppressing non-lysogenic competitors and genes encoding beneficial bacterial phenotypes, creating conditions for continuous bacteriophage amplification, suggesting that bacteriophages contribute to the host’s environmental tolerance. After lysing the host bacterium, lysogenic bacteriophages infecting *S. aureus* release a small number of transducing particles, which “evade” the immune system of the remaining prophage by injecting DNA into the prophage, thereby conferring beneficial genes to the prophage. Consequently, *S. aureus* has been able to continue to reproduce under the pressure of intense antibiotic environments [78]. The bacteriophage interference carries the risk of inducing the evolution of multiple antibiotic-resistant bacteria, and its potential impact on ARG diffusion in microcosms may be underestimated. After 7 h of incubation with *S. aureus* carrying different ARGs in an identical environment, bacteriophage transduction contributes to the generation of bi-resistant bacteria, with one transducing bacteriophage carrying an ARG among 10^8^ newly generated bacteriophages [79]. It has been suggested that ARGs are rare in bacteriophages but widely present in phage–plasmids (P-Ps, nucleic acid sequences of lysogenic bacteriophages not embedded in the host genome that replicate and translocate independently) [80]. Among the four common antibiotic-resistant bacteria (*E. coli*, *Salmonella*, *Acinetobacter baumannii*, and *Klebsiella pneumoniae*), 78.33% of P-Ps carry ARGs, and different types of ARGs are distributed at different loci in the genomes of the P-Ps in response to transposons and their plasticity varies [81]. While these gene transfer processes provide essential drivers for bacteria to adapt to environmental changes, they simultaneously increase the likelihood of ARG proliferation in the environment and in livestock. On the other side, some scholars have combined bacteriophages with CRISPR-Cas technology to provide fresh ideas on the inhibition of ARGs spreading among bacteria. The CRISPR-Cas system is considered to be a suppressor of horizontal gene transfer between bacterial populations. By selectively removing gene content that carries ARGs, the CRISPR-Cas system restores sensitivity to antibiotics in multidrug-resistant *E. faecalis* and significantly inhibits the spread of ARGs in the *E. faecalis* population [82]. Delivery of the modified CRISPR-Cas system to antibiotic-resistant bacterial genomes using lysogenic bacteriophage can kill antibiotic-resistant bacteria, effectively preventing the spread of ARGs between bacteria [83]. The development of bacteriophages containing the CRISPR-Cas9 system is a novel approach to inhibiting the spread of ARGs; however, the CRISPR-Cas9 delivery modality needs to be optimized by substantial research work in order to enhance the efficiency of removing ARGs from antibiotic-resistant bacteria.

### 5.3. The Instability of Bacteriophages

Our current understanding of the relationship between bacteriophages and animal gut microbiota and immunity is extremely limited, and there is instability in the effects of bacteriophage entry into animals. LPSs not only enter the environment with bacterial rupture and disintegration but are also released into the environment during normal bacterial growth and division, inducing toxic effects such as topical inflammation and endotoxin hemolysis [84]. Therefore, bacteriophage application to intestinal microbes requires careful consideration as bacteriophage lysis of Gram-negative bacteria (e.g., *Salmonella*) releases LPSs from the bacterial cell wall into the intestinal lumen, which may induce an inflammatory response [85]. It has been shown that both active and heat-inactivated *E. coli* bacteriophages and bacteriophages targeting the major intestinal commensals (*Mycobacterium avium* and Gram-positive *L. plantarum*), whose internal DNA can be recognized by the intracellular receptor TLR9, activate dendritic cells and deliver antigenic signals to CD4+ T-cells, inducing the synthesis of IFN-γ to aggravate the inflammatory response in the colon [86]. In a rat model, bacteriophage cocktail supplementation via drinking water significantly increased plasma endotoxin concentrations, serum TNF-α, IL-1β, and IL-6 concentrations, and the urinary lactulose/mannitol ratio [87]. Whether the changes in these indicators are caused by damage to the gut barrier or by bacteriophages reconfiguring the bacterial community is not clear, as the morphology of gut structure and the mRNA expression of tight junction proteins are not detected in that study. While enhancing the intestinal mucosal barrier to prevent the transmucosal transfer of pathogens, bacteriophages may also trigger and induce immune cell responses due to their diversity, and an imbalance in the commensal bacteriophage population induces a variety of chronic immune diseases such as IBD, neurological disorders, and obesity [54,88]. Interestingly, we observed *Caudovirales* bacteriophages maintaining high levels of relative abundance throughout the unbalanced gut, a feature that potentially hints at a correlation between specific phages and disease, but the specific mechanisms remain to be thoroughly investigated [86,89,90]. The reason for the elevated endotoxin concentration in plasma may be the infiltration of high amounts of LPS produced by bacteriophage lysis of host bacteria into the bloodstream during the production of bacteriophage preparations; therefore, endotoxin detoxification and safety evaluation of bacteriophage preparations are vitally important for bacteriophage applications. The scientific guidelines for bacteriophage veterinary drugs issued by the European Medicines Agency clearly regulate the safety and stability of bacteriophage veterinary drug products, including the stability of the active substance, the tolerance of the target animal, the toxicity of repeated doses, and the environmental risk [27]. Whereas Belleghem et al. [91] demonstrated that bacteriophages binding to immune receptors in animals activate both pro-inflammatory immune responses (up-regulation of IL-1α, IL-1β, and TNF-α) and anti-inflammatory immune responses (up-regulation of Interleukin-1 Receptor Antagonist and Suppressor of Cytokine Signaling 3). From the bacteriophage perspective, the subsequent immune response triggered by the bacteriophages tends to protect its host in order to allow for stronger environmental fitness and proliferation opportunities and to remove pathogens in the case of organismal abnormalities. On the other hand, the formulation of the bacteriophage cocktails determines the final application to some extent. Gao et al. [92] designed a mixture including four bacteriophages for the receptors (LPS O antigen, the LPS outer core, the LPS inner core, and the outer membrane proteins BtuB and TolC) on the surface of *Salmonella enterica* serotype enteritidis cells. After placing them in the host bacterial environment and culturing them, it was found that the mixture of bacteriophages could enhance the mutant’s susceptibility to antibiotics and significantly decrease the virulence, showing favorable therapeutic effects compared with the single phage-induced *Salmonella enteritidis* mutant. Pelyuntha et al. [93] also demonstrated that a bacteriophage cocktail consisting of *vB_SenM_P7* and *vB_SenP_P32* significantly inhibited the growth rate of *Salmonella*, broadening the host range and enhancing the lytic efficiency of the bacteriophage at the same time. These studies suggest that a minimum of one component of the bacteriophage cocktail is required to maintain strong inactivating activity against host pathogenic bacteria, thus preventing the growth of pathogens and inhibiting the generation of bacteriophage-resistant genes. The reasonable design of bacteriophage cocktails provides novel ideas for scientifically exploring the safety and stability of bacteriophages in livestock and poultry production.

## 6. Prospects

In the future, we intend to explore the survival conditions of bacteriophages in diverse environments by using culture-omics, combining the mechanism of bacteria–bacteriophage coexistence with culture-based isolation techniques to obtain bacteriophage cocktails that specifically infect bacteria. There are reports on the culture-omics of healthy human intestinal bacteriophages, and researchers have isolated 209 non-redundant bacteriophages infecting 42 species of commensal bacteria in the gut, more than 80% of which belong to the newly discovered taxonomic genus, and identified a family of bacteriophages, *Paboviridae*, with a high prevalence in the human intestinal tract [94]. Therefore, in livestock and poultry farming, we can collect samples from pathological tissues or the environment for different physiological diseases (piglet diarrhea, mastitis in dairy cows, etc.) or the quality of livestock products, and isolate and cultivate bacteriophages specifically infested with pathogens by means of culture-omics. We can also rationally combine bacteriophages for the receptors on the surface of the pathogen, and apply bacteriophage cocktails to reduce the probability of bacteriophage-resistant mutant production since this requires bacteria to evolve multiple orthogonal resistance mechanisms at the same time, which can maximally constrain the growth of the pathogens to solve disease problems [27].

Modification of the bacteriophage genome by applying genetic engineering techniques could lead to more precise treatments. Typically, lysing bacteria with lytic bacteriophages is the simplest and most efficient way, but certain bacteria (*Mycobacterium* spp.) prefer to be infected by some lysogenic bacteriophages [95]. Accordingly, we can break the lysogenic bacteria–bacteriophage coexistence by removing part or all of the repressor genes to promote the bacteriophage transition from a lysogenic to a lytic state. A recent study found that the presence of phage-encoded RNA anti-CRISPRs (Racrs) in *Thiocystis violascens* prophage is capable of interacting specifically with Cas6f and Cas7f, strongly inhibiting the type I-F CRISPR-Cas system, leading to the formation of aberrant CRISPR-Cas subcomplexes and disrupting the bacterial CRISPR-Cas defense system. RacrIF1 is found in prophages that infest multiple bacteria (e.g., Firmicutes and Proteobacteria), suggesting that some bacteriophages possess the potential of silencing bacterial CRISPR systems, and genes encoding Racrs could be transduced into bacteriophage genomes to reduce the risk of ARG dissemination in the future [71]. Moreover, the established functional macro genomic approach DEEPMINE can exchange the tail information of various bacteriophage types to expand the host range of the *T7* phage as well [96]. The application of multiple gene editing techniques helps us to gain a clearer understanding of the interaction mechanism between bacteriophages and bacterial communities, and there are more options for the livestock and poultry industries to achieve bacteriophage industrialization and promotion.

It should be noted that the application effects of bacteriophage cocktails are not only related to the specific formulation but also to the order of application, duration, and quality control of each bacteriophage. Therefore, bacteriophage species should be selected according to realistic situations and their instability should be minimized as much as possible. Finally, there is still a lot of research required before bacteriophage application can be promoted in the farm animal industry.

## 7. Conclusions

In summary, bacteriophages, as a class of living organisms co-evolving with host bacteria, have shed light on the challenges of antibiotic substitution in livestock and poultry production due to their diversity and adaptability. Following a systematic review, we identified the essential roles of bacteriophages in regulating the structure of intestinal flora, participating in immune response, improving intestinal morphology, etc. Meanwhile, we can’t ignore the future challenges in bacteriophage application. If we can fully explore the potential of bacteriophages, these viruses can be used as a favorable tool to solve the looming threat of antibiotic resistance, as well as bring us great ecological and economic benefits.

## Data Availability

Not applicable.

## References

[1] Castanon J.I. (2007). History of the use of antibiotic as growth promoters in european poultry feeds. Poult. Sci..

[2] Lai C.K.C., Ng R.W.Y., Leung S.S.Y., Hui M., Ip M. (2022). Overcoming the rising incidence and evolving mechanisms of antibiotic resistance by novel drug delivery approaches—An overview. Adv. Drug Deliv. Rev..

[3] Sabino Y.N.V., Santana M.F., Oyama L.B., Santos F.G., Moreira A.J.S., Huws S.A., Mantovani H.C. (2019). Characterization of antibiotic resistance genes in the species of the rumen microbiota. Nat. Commun..

[4] Nguyet L.T.Y., Keeratikunakorn K., Kaeoket K., Ngamwongsatit N. (2022). Antibiotic resistant *Escherichia coli* from diarrheic piglets from pig farms in thailand that harbor colistin-resistant mcr genes. Sci. Rep..

[5] Chang Q., Wang W., Regev-Yochay G., Lipsitch M., Hanage W.P. (2015). Antibiotics in agriculture and the risk to human health: How worried should we be?. Evol. Appl..

[6] Xiao Y., Huang R., Wang N., Deng Y., Tan B., Yin Y., Qi M., Wang J. (2022). Ellagic acid alleviates oxidative stress by mediating nrf2 signaling pathways and protects against paraquat-induced intestinal injury in piglets. Antioxidants.

[7] Fu Q., Cui Q., Yang Y., Zhao X., Song X., Wang G., Bai L., Chen S., Tian Y., Zou Y. (2018). Effect of resveratrol dry suspension on immune function of piglets. Evid. Based Complement. Alternat. Med..

[8] Li W., Zhang X., He Z., Chen Y., Li Z., Meng T., Li Y., Cao Y. (2020). In vitro and in vivo antioxidant activity of eucalyptus leaf polyphenols extract and its effect on chicken meat quality and cecum microbiota. Food Res. Int..

[9] Wang H., Wang C., Zou Y., Hu J., Li Y., Cheng Y. (2020). Natural polyphenols in drug delivery systems: Current status and future challenges. Giant.

[10] Luise D., Bosi P., Raff L., Amatucci L., Virdis S., Trevisi P. (2022). *Bacillus* spp. Probiotic strains as a potential tool for limiting the use of antibiotics, and improving the growth and health of pigs and chickens. Front. Microbiol..

[11] Leenay R.T., Vento J.M., Shah M., Martino M.E., Leulier F., Beisel C.L. (2019). Genome editing with crispr-cas9 in lactobacillus plantarum revealed that editing outcomes can vary across strains and between methods. Biotechnol. J..

[12] Wang H., Long W., Chadwick D., Zhang X., Zhang S., Piao X., Hou Y. (2022). Dietary acidifiers as an alternative to antibiotics for promoting pig growth performance: A systematic review and meta-analysis. Anim. Feed. Sci. Technol..

[13] Rahman M.R.T., Fliss I., Biron E. (2022). Insights in the development and uses of alternatives to antibiotic growth promoters in poultry and swine production. Antibiotics.

[14] Shkoporov A.N., Turkington C.J., Hill C. (2022). Mutualistic interplay between bacteriophages and bacteria in the human gut. Nat. Rev. Microbiol..

[15] Chevallereau A., Pons B.J., van Houte S., Westra E.R. (2022). Interactions between bacterial and phage communities in natural environments. Nat. Rev. Microbiol..

[16] Schooley R.T., Biswas B., Gill J.J., Hernandez-Morales A., Lancaster J., Lessor L., Barr J.J., Reed S.L., Rohwer F., Benler S. (2017). Development and use of personalized bacteriophage-based therapeutic cocktails to treat a patient with a disseminated resistant acinetobacter baumannii infection. Antimicrob. Agents Chemother..

[17] Bao J., Wu N., Zeng Y., Chen L., Li L., Yang L., Zhang Y., Guo M., Li L., Li J. (2020). Non-active antibiotic and bacteriophage synergism to successfully treat recurrent urinary tract infection caused by extensively drug-resistant *Klebsiella pneumoniae*. Emerg. Microbes Infect..

[18] Cheng R., Li X., Jiang L., Gong L., Geslin C., Shao Z. (2022). Virus diversity and interactions with hosts in deep-sea hydrothermal vents. Microbiome.

[19] Li Z., Pan D., Wei G., Pi W., Zhang C., Wang J.H., Peng Y., Zhang L., Wang Y., Hubert C.R.J. (2021). Deep sea sediments associated with cold seeps are a subsurface reservoir of viral diversity. ISME J..

[20] Suttle C.A. (2005). Viruses in the sea. Nature.

[21] Mushegian A.R. (2020). Are there 1031 virus particles on earth, or more, or fewer?. J. Bacteriol..

[22] Dedrick R.M., Guerrero-Bustamante C.A., Garlena R.A., Russell D.A., Ford K., Harris K., Gilmour K.C., Soothill J., Jacobs-Sera D., Schooley R.T. (2019). Engineered bacteriophages for treatment of a patient with a disseminated drug-resistant *Mycobacterium abscessus*. Nat. Med..

[23] Yan M., Pratama A.A., Somasundaram S., Li Z., Jiang Y., Sullivan M.B., Yu Z. (2023). Interrogating the viral dark matter of the rumen ecosystem with a global virome database. Nat. Commun..

[24] Tao S., Tao H., Li J., Wei H. (2022). Landscapes of enteric virome signatures in early-weaned piglets. Microbiol. Spectr..

[25] Blanco-Picazo P., Gomez-Gomez C., Aguilo-Castillo S., Fernandez-Orth D., Cerda-Cuellar M., Muniesa M., Rodriguez-Rubio L. (2022). Chicken liver is a potential reservoir of bacteriophages and phage-derived particles containing antibiotic resistance genes. Microb. Biotechnol..

[26] Le S., Chen L.G., Zhu T.Y. (2023). Chinese expert recommendation on phage therapy in the clinical practice. Chin. J. Infect. Dis..

[27] Quality, Safety and Efficacy of Bacteriophages as Veterinary Medicines—Scientific Guideline. https://www.ema.europa.eu/en/quality-safety-efficacy-bacteriophages-veterinary-medicines-scientific-guideline.

[28] Hampton H.G., Watson B.N.J., Fineran P.C. (2020). The arms race between bacteria and their phage foes. Nature.

[29] Pires D.P., Melo L.D.R., Azeredo J. (2021). Understanding the complex phage-host interactions in biofilm communities. Annu. Rev. Virol..

[30] Federici S., Kredo-Russo S., Valdés-Mas R., Kviatcovsky D., Weinstock E., Matiuhin Y., Silberberg Y., Atarashi K., Furuichi M., Oka A. (2022). Targeted suppression of human ibd-associated gut microbiota commensals by phage consortia for treatment of intestinal inflammation. Cell.

[31] Zeng Y., Wang Z., Zou T., Chen J., Li G., Zheng L., Li S., You J. (2021). Bacteriophage as an alternative to antibiotics promotes growth performance by regulating intestinal inflammation, intestinal barrier function and gut microbiota in weaned piglets. Front. Vet. Sci..

[32] Kim J.S., Hosseindoust A., Lee S.H., Choi Y.H., Kim M.J., Lee J.H., Kwon I.K., Chae B.J. (2017). Bacteriophage cocktail and multi-strain probiotics in the feed for weanling pigs: Effects on intestine morphology and targeted intestinal coliforms and clostridium. Animal.

[33] Kim K.H., Ingale S.L., Kim J.S., Lee S.H., Lee J.H., Kwon I.K., Chae B.J. (2014). Bacteriophage and probiotics both enhance the performance of growing pigs but bacteriophage are more effective. Anim. Feed Sci. Technol..

[34] Xue C., Goldenfeld N. (2017). Coevolution maintains diversity in the stochastic “kill the winner” model. Phys. Rev. Lett..

[35] Grabowski L., Wegrzyn G., Wegrzyn A., Podlacha M. (2022). Phage therapy vs. The use of antibiotics in the treatment of salmonella-infected chickens: Comparison of effects on hematological parameters and selected biochemical markers. Antibiotics.

[36] Lee M., Hosseindoust A., Oh S., Ko H., Cho E., Sa S., Kim Y., Choi J., Kim J. (2021). Impact of an anti-salmonella. Typhimurium bacteriophage on intestinal microbiota and immunity status of laying hens. J. Anim. Physiol. Anim. Nutr..

[37] Upadhaya S.D., Ahn J.M., Cho J.H., Kim J.Y., Kang D.K., Kim S.W., Kim H.B., Kim I.H. (2021). Bacteriophage cocktail supplementation improves growth performance, gut microbiome and production traits in broiler chickens. J. Anim. Sci. Biotechnol..

[38] Auffret M.D., Dewhurst R.J., Duthie C.A., Rooke J.A., John Wallace R., Freeman T.C., Stewart R., Watson M., Roehe R. (2017). The rumen microbiome as a reservoir of antimicrobial resistance and pathogenicity genes is directly affected by diet in beef cattle. Microbiome.

[39] Sarrami Z., Sedghi M., Mohammadi I., Kim W.K., Mahdavi A.H. (2022). Effects of bacteriophage supplement on the growth performance, microbial population, and pgc-1alpha and tlr4 gene expressions of broiler chickens. Sci. Rep..

[40] Chatterjee A., Willett J.L.E., Dunny G.M., Duerkop B.A. (2021). Phage infection and sub-lethal antibiotic exposure mediate *Enterococcus faecalis* type vii secretion system dependent inhibition of bystander bacteria. PLoS Genet..

[41] Hsu B.B., Gibson T.E., Yeliseyev V., Liu Q., Lyon L., Bry L., Silver P.A., Gerber G.K. (2019). Dynamic modulation of the gut microbiota and metabolome by bacteriophages in a mouse model. Cell Host Microbe.

[42] Vandenheuvel D., Lavigne R., Brussow H. (2015). Bacteriophage therapy: Advances in formulation strategies and human clinical trials. Annu. Rev. Virol..

[43] Ge H., Hu M., Zhao G., Du Y., Xu N., Chen X., Jiao X. (2020). The “fighting wisdom and bravery” of tailed phage and host in the process of adsorption. Microbiol. Res..

[44] Pires D.P., Oliveira H., Melo L.D., Sillankorva S., Azeredo J. (2016). Bacteriophage-encoded depolymerases: Their diversity and biotechnological applications. Appl. Microbiol. Biotechnol..

[45] Wandro S., Ghatbale P., Attai H., Hendrickson C., Samillano C., Suh J., Dunham S.J.B., Pride D.T., Whitesona K. (2022). Phage cocktails constrain the growth of enterococcus. Msystems.

[46] Lee H.Y., Biswas D., Ahn J. (2015). In-vitro adhesion and invasion properties of *Salmonella typhimurium* competing with bacteriophage in epithelial cells and chicken macrophages. Rev. Bras. Ciência Avícola.

[47] Saez A.C., Zhang J.Y., Rostagno M.H., Ebner P.D. (2011). Direct feeding of microencapsulated bacteriophages to reduce salmonella colonization in pigs. Foodborne Pathog. Dis..

[48] Seo B.-J., Song E.-T., Lee K., Kim J.-W., Jeong C.-G., Moon S.-H., Son J.S., Kang S.H., Cho H.-S., Jung B.Y. (2018). Evaluation of the broad-spectrum lytic capability of bacteriophage cocktails against various salmonella serovars and their effects on weaned pigs infected with *Salmonella typhimurium*. J. Vet. Med. Sci..

[49] Guo M., Gao Y., Xue Y., Liu Y., Zeng X., Cheng Y., Ma J., Wang H., Sun J., Wang Z. (2021). Bacteriophage cocktails protect dairy cows against mastitis caused by drug resistant *Escherichia coli* infection. Front. Cell Infect. Microbiol..

[50] Smith H.W., Huggins M.B. (1983). Effectiveness of phages in treating experimental *Escherichia coli* diarrhoea in calves, piglets and lambs. J. Gen. Microbiol..

[51] Burmeister A.R., Fortier A., Roush C., Lessing A.J., Bender R.G., Barahman R., Grant R., Chan B.K., Turner P.E. (2020). Pleiotropy complicates a trade-off between phage resistance and antibiotic resistance. Proc. Natl. Acad. Sci. USA.

[52] Miernikiewicz P., Dabrowska K. (2022). Endocytosis of bacteriophages. Curr. Opin. Virol..

[53] Nguyen S., Baker K., Padman B.S., Patwa R., Dunstan R.A., Weston T.A., Schlosser K., Bailey B., Lithgow T., Lazarou M. (2017). Bacteriophage transcytosis provides a mechanism to cross epithelial cell layers. mBio.

[54] Barr J.J., Auro R., Furlan M., Whiteson K.L., Erb M.L., Pogliano J., Stotland A., Wolkowicz R., Cutting A.S., Doran K.S. (2013). Bacteriophage adhering to mucus provide a non-host-derived immunity. Proc. Natl. Acad. Sci. USA.

[55] Yan A., Butcher J., Schramm L., Mack D.R., Stintzi A. (2023). Multiomic spatial analysis reveals a distinct mucosa-associated virome. Gut Microbes.

[56] Lee S., Hosseindoust A., Goel A., Choi Y., Kwon I.K., Chae B. (2016). Effects of dietary supplementation of bacteriophage with or without zinc oxide on the performance and gut development of weanling pigs. Ital. J. Anim. Sci..

[57] Hosseindoust A.R., Lee S.H., Kim J.S., Choi Y.H., Noh H.S., Lee J.H., Jha P.K., Kwon I.K., Chae B.J. (2017). Dietary bacteriophages as an alternative for zinc oxide or organic acids to control diarrhoea and improve the performance of weanling piglets. Veterinární Medicína.

[58] Zhao H., Li Y., Lv P., Huang J., Tai R., Jin X., Wang J., Wang X. (2022). Salmonella phages affect the intestinal barrier in chicks by altering the composition of early intestinal flora: Association with time of phage use. Front. Microbiol..

[59] Koskella B., Brockhurst M.A. (2014). Bacteria-phage coevolution as a driver of ecological and evolutionary processes in microbial communities. FEMS Microbiol. Rev..

[60] Labrie S.J., Samson J.E., Moineau S. (2010). Bacteriophage resistance mechanisms. Nat. Rev. Microbiol..

[61] Hao G., Yuan C., Shu R., Jia Y., Zhao S., Xie S., Liu M., Zhou H., Sun S., Wang H. (2021). O-antigen serves as a two-faced host factor for bacteriophage njs1 infecting nonmucoid *Klebsiella pneumoniae*. Microb. Pathog..

[62] Chatterjee A., Johnson C.N., Luong P., Hullahalli K., McBride S.W., Schubert A.M., Palmer K.L., Carlson P.E., Duerkop B.A. (2019). Bacteriophage resistance alters antibiotic-mediated intestinal expansion of enterococci. Infect. Immun..

[63] Lu M.-J., Henning U. (1994). Superinfection exclusion by t-even-type coliphages. Trends Microbiol..

[64] Vasu K., Nagaraja V. (2013). Diverse functions of restriction-modification systems in addition to cellular defense. Microbiol. Mol. Biol. Rev..

[65] Barrangou R., Fremaux C., Deveau H., Richards M., Boyaval P., Moineau S., Romero D.A., Horvath P. (2007). Crispr provides acquired resistance against viruses in prokaryotes. Science.

[66] Blasco L., López-Hernández I., Rodríguez-Fernández M., Pérez-Florido J., Casimiro-Soriguer C.S., Djebara S., Merabishvili M., Pirnay J.-P., Rodríguez-Baño J., Tomás M. (2023). Case report: Analysis of phage therapy failure in a patient with a *Pseudomonas aeruginosa* prosthetic vascular graft infection. Front. Med..

[67] Meyer J.R., Dobias D.T., Weitz J.S., Barrick J.E., Quick R.T., Lenski R.E. (2012). Repeatability and contingency in the evolution of a key innovation in phage lambda. Science.

[68] Piya D., Vara L., Russell W.K., Young R., Gill J.J. (2017). The multicomponent antirestriction system of phage p1 is linked to capsid morphogenesis. Mol. Microbiol..

[69] Guo T.W., Bartesaghi A., Yang H., Falconieri V., Rao P., Merk A., Eng E.T., Raczkowski A.M., Fox T., Earl L.A. (2017). Cryo-em structures reveal mechanism and inhibition of DNA targeting by a crispr-cas surveillance complex. Cell.

[70] Huiting E., Cao X., Ren J., Athukoralage J.S., Luo Z., Silas S., An N., Carion H., Zhou Y., Fraser J.S. (2023). Bacteriophages inhibit and evade cgas-like immune function in bacteria. Cell.

[71] Camara-Wilpert S., Mayo-Munoz D., Russel J., Fagerlund R.D., Madsen J.S., Fineran P.C., Sorensen S.J., Pinilla-Redondo R. (2023). Bacteriophages suppress crispr-cas immunity using rna-based anti-crisprs. Nature.

[72] Abdelrahman F., Easwaran M., Daramola O.I., Ragab S., Lynch S., Oduselu T.J., Khan F.M., Ayobami A., Adnan F., Torrents E. (2021). Phage-encoded endolysins. Antibiotics.

[73] Jun S.Y., Jang I.J., Yoon S., Jang K., Yu K.-S., Cho J.Y., Seong M.-W., Jung G.M., Yoon S.J., Kang S.H. (2017). Pharmacokinetics and tolerance of the phage endolysin-based candidate drug sal200 after a single intravenous administration among healthy volunteers. Antimicrob. Agents Chemother..

[74] Jun S.Y., Jung G.M., Yoon S.J., Oh M.-D., Choi Y.-J., Lee W.J., Kong J.-C., Seol J.G., Kang S.H. (2013). Antibacterial properties of a pre-formulated recombinant phage endolysin, sal-1. Int. J. Antimicrob. Agents.

[75] Blair J.M., Webber M.A., Baylay A.J., Ogbolu D.O., Piddock L.J. (2015). Molecular mechanisms of antibiotic resistance. Nat. Rev. Microbiol..

[76] Hitch T.C.A., Thomas B.J., Friedersdorff J.C.A., Ougham H., Creevey C.J. (2018). Deep sequence analysis reveals the ovine rumen as a reservoir of antibiotic resistance genes. Environ. Pollut..

[77] Horvath P., Barrangou R. (2010). Crispr/cas, the immune system of bacteria and archaea. Science.

[78] Haaber J., Leisner J.J., Cohn M.T., Catalan-Moreno A., Nielsen J.B., Westh H., Penades J.R., Ingmer H. (2016). Bacterial viruses enable their host to acquire antibiotic resistance genes from neighbouring cells. Nat. Commun..

[79] Leclerc Q.J., Wildfire J., Gupta A., Lindsay J.A., Knight G.M. (2022). Growth-dependent predation and generalized transduction of antimicrobial resistance by bacteriophage. Msystems.

[80] Pfeifer E., Moura de Sousa J.A., Touchon M., Rocha E.P.C. (2021). Bacteria have numerous distinctive groups of phage-plasmids with conserved phage and variable plasmid gene repertoires. Nucleic Acids Res..

[81] Pfeifer E., Bonnin R.A., Rocha E.P.C. (2022). Phage-plasmids spread antibiotic resistance genes through infection and lysogenic conversion. mBio.

[82] Price V.J., Huo W., Sharifi A., Palmer K.L. (2016). Crispr-cas and restriction-modification act additively against conjugative antibiotic resistance plasmid transfer in *Enterococcus faecalis*. mSphere.

[83] Yosef I., Manor M., Kiro R., Qimron U. (2015). Temperate and lytic bacteriophages programmed to sensitize and kill antibiotic-resistant bacteria. Proc. Natl. Acad. Sci. USA.

[84] Otto L., Chris G., Ernst T.R. (1982). Endotoxins of gram-negative bacteria. Pharmacol. Ther..

[85] Ling H., Lou X., Luo Q., He Z., Sun M., Sun J. (2022). Recent advances in bacteriophage-based therapeutics: Insight into the post-antibiotic era. Acta Pharm. Sin. B.

[86] Gogokhia L., Buhrke K., Bell R., Hoffman B., Brown D.G., Hanke-Gogokhia C., Ajami N.J., Wong M.C., Ghazaryan A., Valentine J.F. (2019). Expansion of bacteriophages is linked to aggravated intestinal inflammation and colitis. Cell Host Microbe.

[87] Tetz G.V., Ruggles K.V., Zhou H., Heguy A., Tsirigos A., Tetz V. (2017). Bacteriophages as potential new mammalian pathogens. Sci. Rep..

[88] Tisza M.J., Buck C.B. (2021). A catalog of tens of thousands of viruses from human metagenomes reveals hidden associations with chronic diseases. Proc. Natl. Acad. Sci. USA.

[89] Norman J.M., Handley S.A., Baldridge M.T., Droit L., Liu C.Y., Keller B.C., Kambal A., Monaco C.L., Zhao G., Fleshner P. (2015). Disease-specific alterations in the enteric virome in inflammatory bowel disease. Cell.

[90] Zuo T., Lu X.-J., Zhang Y., Cheung C.P., Lam S., Zhang F., Tang W., Ching J.Y.L., Zhao R., Chan P.K.S. (2019). Gut mucosal virome alterations in ulcerative colitis. Gut.

[91] Van Belleghem J.D., Clement F., Merabishvili M., Lavigne R., Vaneechoutte M. (2017). Pro- and anti-inflammatory responses of peripheral blood mononuclear cells induced by staphylococcus aureus and *Pseudomonas aeruginosa* phages. Sci. Rep..

[92] Gao G.F., Ji H., Wang L., Li X. (2022). Fitness trade-offs in phage cocktail-resistant salmonella enterica serovar enteritidis results in increased antibiotic susceptibility and reduced virulence. Microbiol. Spectr..

[93] Pelyuntha W., Vongkamjan K. (2022). Combined effects of salmonella phage cocktail and organic acid for controlling salmonella enteritidis in chicken meat. Food Control.

[94] Shen J., Zhang J., Mo L., Li Y., Li Y., Li C., Kuang X., Tao Z., Qu Z., Wu L. (2023). Large-scale phage cultivation for commensal human gut bacteria. Cell Host Microbe.

[95] Hatfull G.F. (2020). Actinobacteriophages: Genomics, dynamics, and applications. Annu. Rev. Virol..

[96] Apjok G., Szamel M., Christodoulou C., Seregi V., Vasarhelyi B.M., Stirling T., Eszenyi B., Sari T., Vidovics F., Nagrand E. (2023). Characterization of antibiotic resistomes by reprogrammed bacteriophage-enabled functional metagenomics in clinical strains. Nat. Microbiol..

